# A Novel Boron Lipid to Modify Liposomal Surfaces for Boron Neutron Capture Therapy

**DOI:** 10.3390/cells10123421

**Published:** 2021-12-05

**Authors:** Makoto Shirakawa, Alexander Zaboronok, Kei Nakai, Yuhki Sato, Sho Kayaki, Tomonori Sakai, Takao Tsurubuchi, Fumiyo Yoshida, Takashi Nishiyama, Minoru Suzuki, Hisao Tomida, Akira Matsumura

**Affiliations:** 1 Department of Pharmaceutical Sciences, Fukuyama University, 1-985 Higashimuracho-Sanzo, Fukuyama, Hiroshima 729-0292, Japan; s-yuhki@fukuyama-u.ac.jp (Y.S.); p7117035@fukuyama-u.ac.jp (S.K.); p7117057@fukuyama-u.ac.jp (T.S.); t_nishiyama@fukuyama-u.ac.jp (T.N.); tomidahi@hi3.enjoy.ne.jp (H.T.); 2 Department of Radiation Oncology, Faculty of Medicine, University of Tsukuba, 1-1-1 Tennodai, Tsukuba 305-8575, Ibaraki, Japan; knakai@pmrc.tsukuba.ac.jp (K.N.); matsumura.akira.ft@alumni.tsukuba.ac.jp (A.M.); 3 Department of Neurosurgery, Faculty of Medicine, University of Tsukuba, 1-1-1 Tennodai, Tsukuba 305-8575, Japan; a.zaboronok@md.tsukuba.ac.jp (A.Z.); t-tsurubuchi@md.tsukuba.ac.jp (T.T.); yoshida.fumiyo.ff@u.tsukuba.ac.jp (F.Y.); 4 Institute for Integrated Radiation and Nuclear science, Kyoto University, 2 Asashiro-Nishi, Kumatori-cho, Sennan-gun, Osaka 590-0494, Japan; suzuki.minoru.3x@kyoto-u.ac.jp (M.S.); 5 Ibaraki Prefectural University of Health Sciences, 4669-2 Amicho, Inashiki 300-0394, Ibaraki, Japan

**Keywords:** boron neutron capture therapy, boron lipid, liposomes, boron drug

## Abstract

Boron neutron capture therapy (BNCT) is a cancer treatment with clinically demonstrated efficacy using boronophenylalanine (BPA) and sodium mercaptododecaborate (BSH). However, tumor tissue selectivity of BSH and retention of BPA in tumor cells is a constant problem. To ensure boron accumulation and retention in tumor tissues, we designed a novel polyethylene glycol (PEG)-based boron-containing lipid (PBL) and examined the potency of delivery of boron using novel PBL-containing liposomes, facilitated by the enhanced permeability and retention (EPR) effect. PBL was synthesized by the reaction of distearoylphosphoethanolamine and BSH linked by PEG with Michael addition while liposomes modified using PBL were prepared from the mixed lipid at a constant molar ratio. In this manner, novel boron liposomes featuring BSH in the liposomal surfaces, instead of being encapsulated in the inner aqueous phase or incorporated in the lipid bilayer membrane, were prepared. These PBL liposomes also carry additional payload capacity for more boron compounds (or anticancer agents) in their inner aqueous phase. The findings demonstrated that PBL liposomes are promising candidates to effect suitable boron accumulation for BNCT.

## 1. Introduction

Boron neutron capture therapy (BNCT) has attracted considerable attention as a cancer treatment that does not significantly reduce patient quality of life. To ensure the effectiveness of BNCT, it is necessary to accumulate adequate boron within the tumor, and methods to realize this aspect are being actively studied.

In particular, boron delivery systems (BDS) based on drug-delivery systems (DDS) have emerged as promising tools [1] and technologies, such as liposomal packaging, are widely used as DDS materials owing to the convenience of controlling the size and electric potential. Ligand modification (e.g., carbohydrate chains, peptides, and antibodies) in the case of liposomes can be easily realized while the cytotoxicity of liposomal materials is extremely low. In this context, by wrapping boron in liposomes, the effectiveness of BNCT can likely be enhanced.

Various boron liposomes have been developed to date using two key approaches, the first of which involves the encapsulation of boron compounds in liposomes. Yanagie et al. first reported the encapsulation of sodium mercaptododecaborate (BSH) in liposomes conjugated with monoclonal antibodies [2,3] and Hawthorne et al. developed a polyethylene glycol-modified liposome (PEG-liposome) that encapsulated boron compounds [4,5]. Moreover, Maruyama et al. demonstrated that boron liposomes modified with transferrin are highly effective candidates for use in BNCT [6]. The second approach involves the incorporation of lipid-conjugated boron in the liposomal membrane as seen in a report from Hawthorne et al., where they developed a lipid analog with a single carbon chain and a hydrophilic group of nido-carborane [7]. The liposomes prepared using this lipid were used in a BDS for treating cancer-bearing mice. In a similar fashion, Nakamura et al. developed a lipid analog with a double carbon chain and a hydrophilic group of nido-carborane [8] as well as a similar base chain with a hydrophilic group of closo-dodecaborate [9]. The liposomes prepared using this lipid exhibited low toxicity and a higher intratumoral boron concentration.

However, in the case of these manufactured liposomes, the membrane may be destabilized from the high ionic concentration and osmotic pressure. Furthermore, the abovementioned two strategies limit the development of novel drugs that may include additional boron groups.

To overcome these limitations, we propose a novel drug-delivery strategy based on the outer layer of the liposome membrane. Specifically, we synthesized a phospholipid derivative as a novel boron lipid, called polyethylene glycol and boron-cluster-modified lipid (PBL), and prepared liposomes with it. The boron groups of the lipid were located in the outer layer of the liposome and did not interfere with the liposomal membrane or its internal aqueous phase. Herein, we report on the results of the synthesis of PBL and the properties of the liposomes prepared using this novel lipid.

## 2. Materials and Methods

### 2.1. Chemicals and Lipids

Distearoylphosphatidylcholine (DSPC) (COATSOME MC-8080), 1, 2-distearoyl-sn-glycero-3-phosphoethanolamine-poly(ethylene glycol) (DSPE-PEG) (SUNBRIGHT DSPE-020CN), and N-[(3-maleimide-1-oxopropyl)aminopropyl polyethylene glycol-carbamyl] distearoylphosphatidyl-ethanolamine (DSPE-PEG-MAL) (SUNBRIGHT DSPE-020MA) were purchased from NOF Co. (Tokyo, Japan). Cholesterol was purchased from Wako (Osaka, Japan). Sodium mercaptododecaborate, Na_2_B_12_H_11_SH (BSH, Fw = 210.25, ^10^B enriched > 99%) was purchased from KATCHEM (Praha, Czech Republic). All other chemicals were of the highest commercially available grade.

### 2.2. Synthesis of PBL

**Scheme 1:**
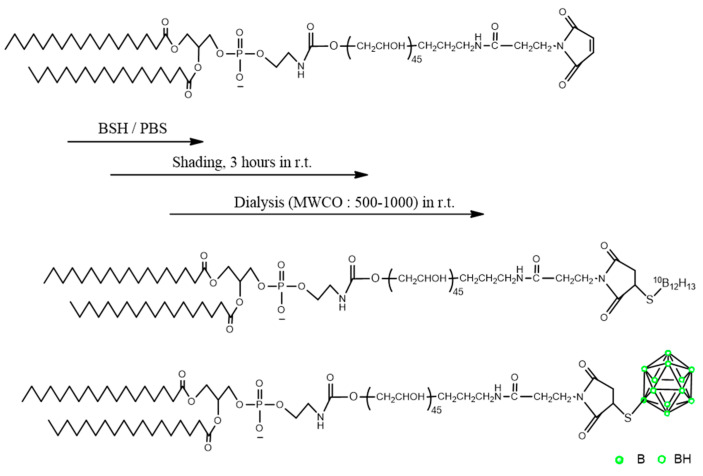
Synthesis and chemical structure of PBL.

PBL was synthesized by the reaction of DSPE-PEG-MAL and BSH. The synthesis of PBL is illustrated in Scheme 1. DSPE-PEG-MAL (120 mg), BSH (40 mg), and phosphate-buffered saline (PBS) (pH = 7.0) were mixed. The resulting solution was stirred at room temperature (18–28 °C) for 3 h and the reaction was allowed to occur, and its progress was monitored using the high-performance liquid chromatography (HPLC) technique. The crude product was obtained, dialyzed to enhance purity (MWCO: 500–1000), and then freeze-dried to obtain the final product. The dialysis was performed for 12 h under room temperature and shading while changing the external solution (Milli-Q water) every 2 h.

### 2.3. Preparation of Liposome-Modified PBL

Bare liposomes were prepared using DSPC and cholesterol (1:1, molar ratio) by using the lipid-film method [10]. Boron-liposome-modified PBL (PBL liposomes) were prepared by coupling PBL (1% to 5% for the total lipid) to the bare liposomes via post-insertion methods [11]. In particular, PBL dissolved in PBS was added to the bare liposomes, and the mixture was incubated for 1 h at 60 °C. While preparing the PBL liposomes, the lipid concentration was set as 10 mg/mL. The resulting liposome solution was extruded through a polycarbonate membrane with a pore size of 100 nm. A portion of the solution was subjected to size-exclusion chromatography (Sepharose CL-4B column), thereby separating the liposome, micelle, and monomolecular fractions in order [12]. The boron concentrations of each fraction were analyzed through inductively coupled plasma-optical emission spectrometry (ICP-OES, SPS5100, SII, Japan), and the PBL incorporation efficiency and boron content in the liposomes were determined. The chemical structure of PBL was drawn using ChemDraw^®^ software version 12.0 (Perkinelmer Informatics, Inc., Waltham, MA, USA).

### 2.4. Physical Properties of Liposomes

After size-exclusion chromatography, each liposome fraction was diluted 10 times using distilled water, and the particle size and zeta potential were determined via dynamic light scattering on a Zetasizer Nano ZS (ZEN3600, Malvern, UK). In addition, the PBL liposomes were observed using a transmission electron microscope (TEM; JEOL JEM-2000EX, Hanaichi Ultra Structure Research Institute, Aichi Japan.) at an accelerating voltage of 100 kV after the liposomes were dispersed onto a 400-mesh, carbon film-supported Cu grid (NEM, Tokyo, Japan) and negative stained by phosphotungstic acid.

Furthermore, the stability of the drug formulation was examined by observing the detachment of PBL from the liposomes in fetal bovine serum at 37 °C—which can be considered as a model of blood—for 48 h. PBL liposomes were added to the medium and chronologically fractionated with stirring. The resulting product was subjected to size-exclusion chromatography. The monomolecular fractions were evaluated using ICP-OES to identify the degree of PBL detachment.

## 3. Results

### 3.1. Identification of PBL

**Figure 1:**
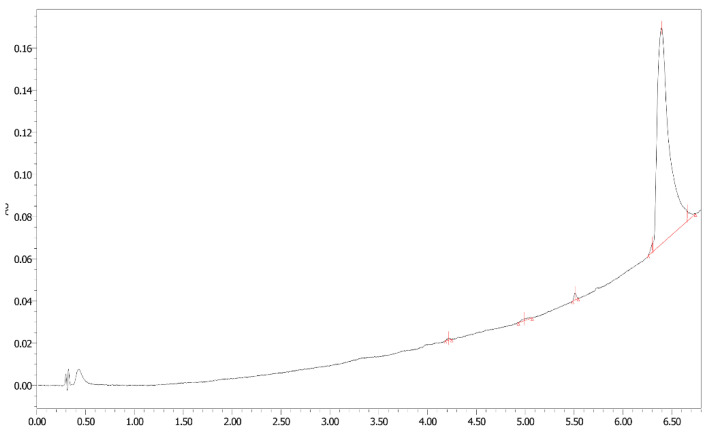
UPLC chromatograms highlighting the purification of PBL by dialysis.

PBL synthesis yield and purity of the product were satisfactory. To confirm purity, analytical ultra-performance liquid chromatography (UPLC) was performed using an ACQUITY UPLC^®^ 1.7 μm BEH300 C4 column (2.1 mm × 50 mm, Waters Corp., Milford, MA, USA) and ACQUITY^®^ UPLC H-Class system (Waters Corp., Milford, MA, USA) in a water (0.1% trifluoroacetic acid; TFA)/acetonitrile (0.1% trifluoroacetic acid) gradient at a flow rate of 0.5 mL/min (95/5 to 20/80 over 7 min). The yield was greater than 64%, and PBL purities were > 96% (Figure 1).

**Figure 2:**
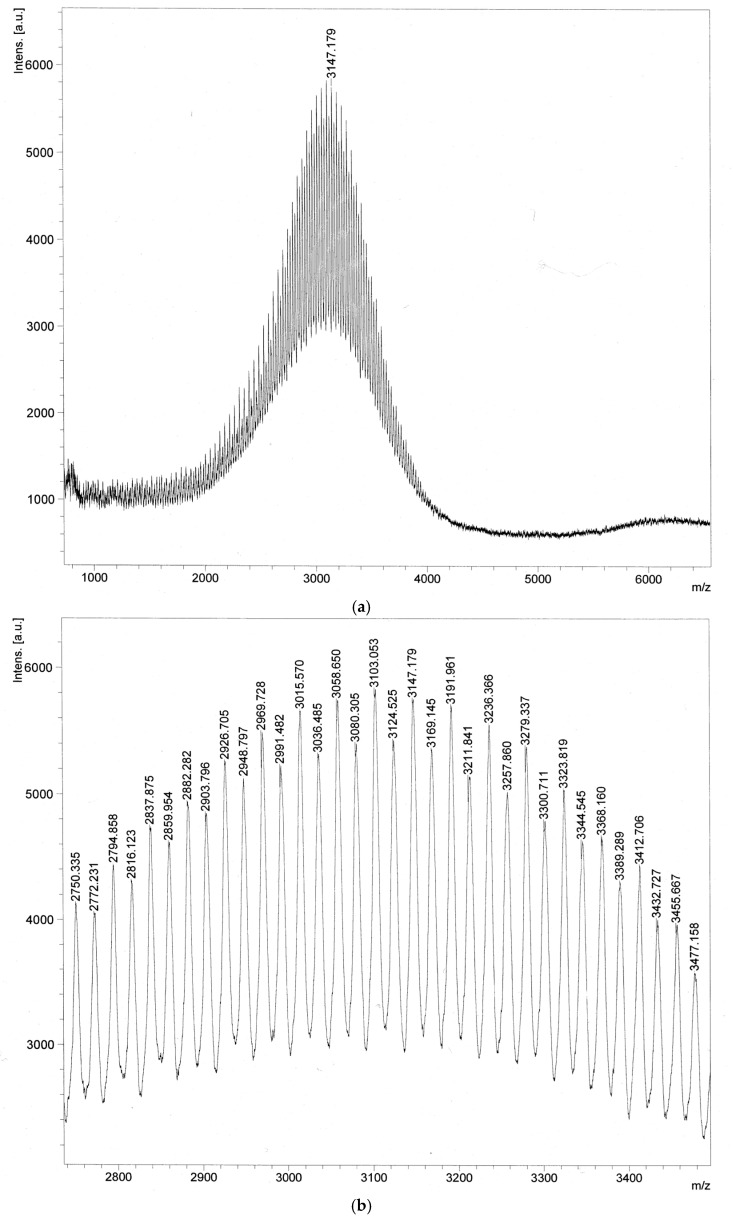
MALDI-TOF-MS spectra of PBL: (**a**) General view; (**b**) enlarged view. Signal intensities are given in arbitrary units [a.u.].

Matrix-assisted laser desorption/ionization-time of flight mass spectrometry (MALDI-TOF-MS) spectra were obtained using an AXIMA^®^ Confidence system (Shimadzu Corp., Kyoto, Japan). The matrix involved α-cyano-4-hydroxycinnamic acid that was dissolved into water containing 0.1% TFA/acetonitrile (1:1). The samples were mixed with the matrix solution in a volume ratio of 1:1 with a final concentration of 0.5 mg/mL. The PBL exact mass was 3146, and a peak of m/z 3147 M + H^+^ was observed (Figure 2a). These spectra indicated a difference of 44 in adjacent peaks, corresponding to one unit (-CH_2_CH_2_O-) of PEG (Figure 2b).

**Figure 3:**
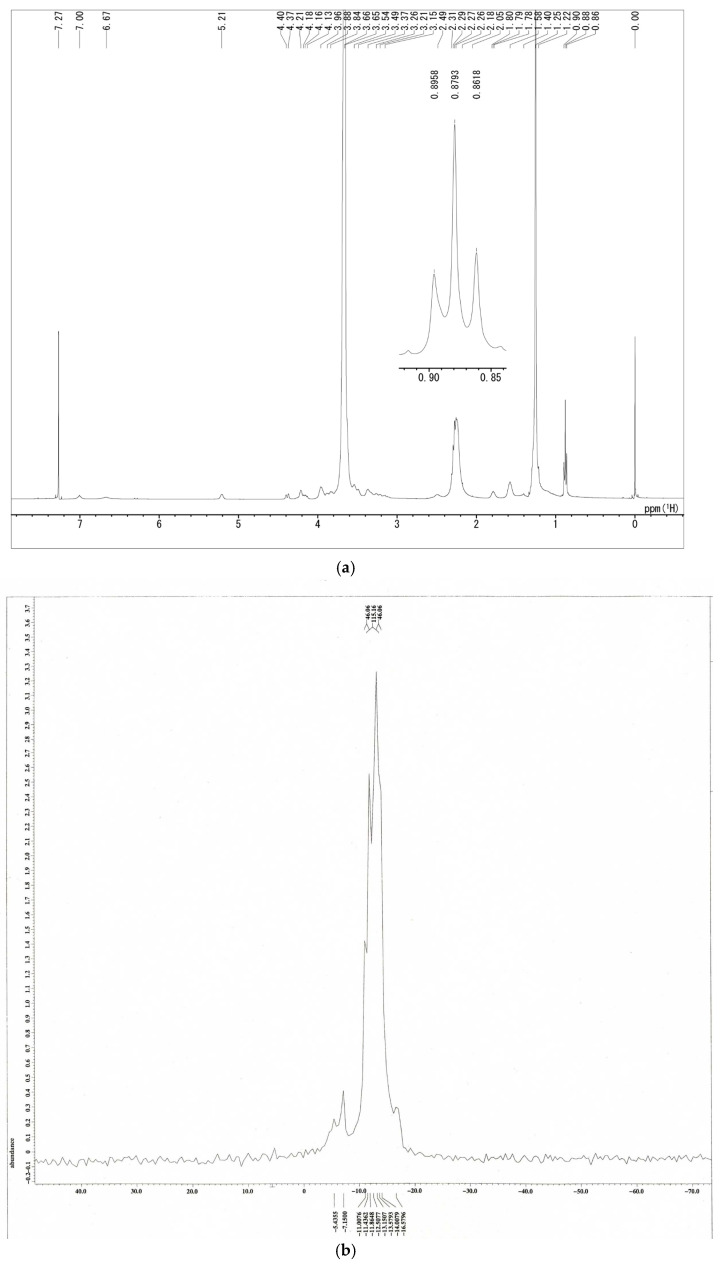
NMR spectra of PBL: (**a**) ^1^H-NMR spectra; (**b**) ^10^B-NMR spectra.

Further, proton nuclear magnetic resonance (^1^H-NMR) spectra were obtained using ECZ400S (JEOL Corp., Tokyo, Japan) and the characteristic structure of PBL was confirmed (Figure 3a). The samples were dissolved in chloroform-*d* at 5 mg/mL. The stearyl moiety of the synthesized PBL exhibited the correct proton ratio to methyl signal at 0.88 ppm at the end of the alkyl chain (6H, as the characteristic signal of lipid). ^10^B-NMR spectra were obtained using JMN-AL400 (JEOL Corp., Tokyo, Japan) and boron cluster moieties of synthesized PBL exhibited this boron signal (Figure 3b).

### 3.2. Incorporation Efficiency of PBL and Characterization of PBL Liposomes

The incorporation efficiency of PBL in the liposomes and properties of these liposomes were examined. Homogeneous, conventional, bare liposomes, composed of DSPC and cholesterol (1:1, molar ratio), were modified using PBL at concentrations of 1–5% by post-insertion methods.

**Figure 4:**
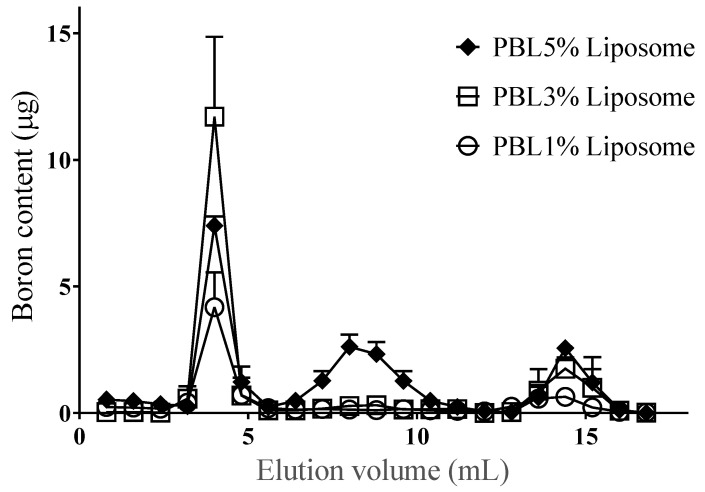
Relationship between the incorporation content of PBL in the liposome with the construction ratio of PBL to liposomes as visualized by size-exclusion chromatography. Each peak represents the following fractions: the first (3.2–4.8 mL) is the liposome peak, the second (4.8–10.4 mL) is the micelle peak, and the third (13.6–15.2 mL) is the monomolecular peak. Data are expressed as means ± S.D. (*n* = 3).

**Figure A1:**
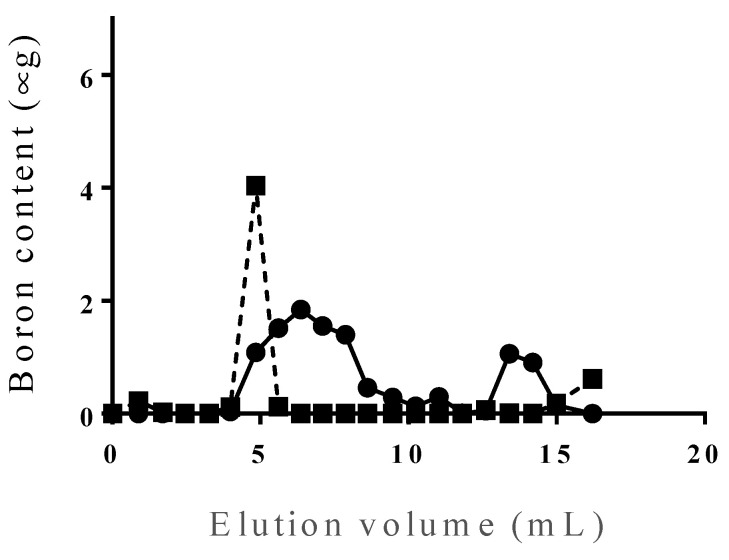
Incorporation of PBL to liposome.

PBL was incorporated only in the liposomal surface (Figure 4). However, when the constituent PBL proportion exceeded 5%, PBL formed micelles, leading to a reduced incorporation efficiency and amount of PBL in the liposomes. Furthermore, when the constituent PBL proportion reached 20%, it could not be incorporated in the liposomes (Figure A1).

**Figure 5:**
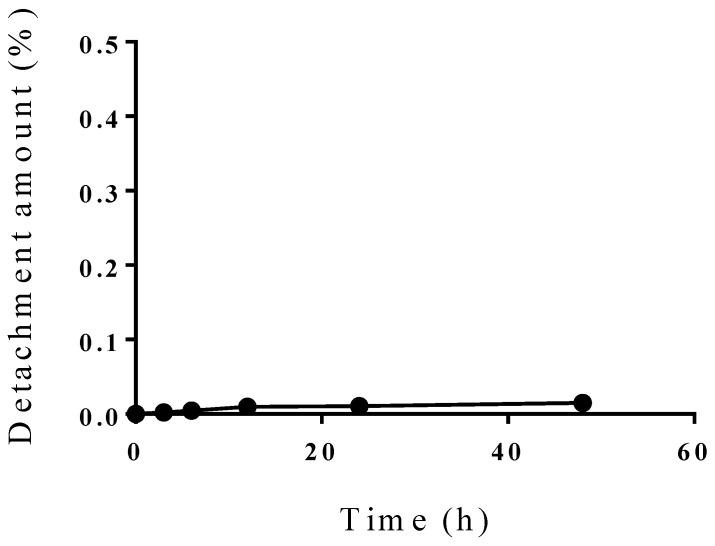
Stability of PBL liposome in a blood model. The stability of the drug formulation was evaluated by measuring the detachment of PBL from the PBL liposomes (PBL content of 5%) for 48 h in fetal bovine serum at 37 °C. Values indicate the total amount of PBL detachment at the indicated time point (●).

The characteristics of the PBL liposomes are summarized in Table 1. Up to a PBL content of 3%, the incorporation efficiency of PBL was approximately 65–70%. When the PBL content was 3%, the amount of PBL incorporated in the liposomes was maximized and the PBL content in the liposomes was 0.1 μmol. Assuming that the molecular weight of liposomes made of phospholipids is 2 million Daltons [13], PBL liposomes with BSH bound to the end of the PEG chain will have about 840 boron atoms (70 PBL molecules) per liposome on their surface. The detachment of PBL from the liposomes was insignificant up to a PBL content of 5% (Figure 5).

**Figure 6:**
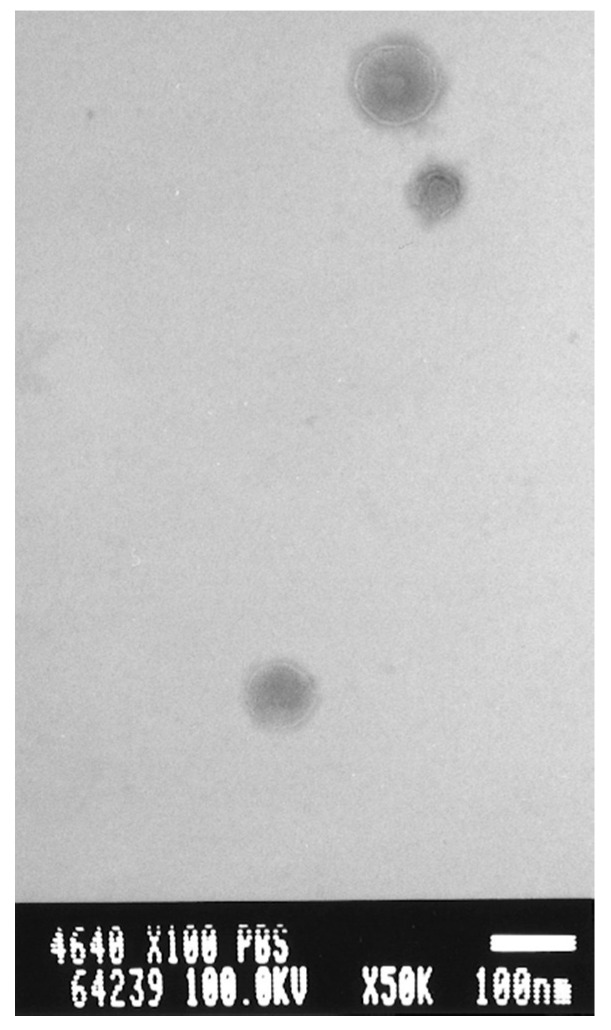
Transmission electron microscopy image of the vesicle formation from the PBL mixture after size-exclusion chromatography.

The PBL liposomes exhibited an anion charge, indicating the presence of PBL on the liposomal surface. The anion charge increased and reached its maximum value at 3% concomitant with increases in the PBL content. In other words, no more PBL could be modified on the liposomal surface. Additionally, the average diameter of the liposomes in all conditions was 150 ± 20 nm. The formation of PBL liposomes was analyzed through transmission electron microscopy based on the negative staining method. The liposomes appeared as unilamellar particles with a diameter of approximately 100 nm (Figure 6).

## 4. Discussion

Liposomes as boron carriers for BNCT have been synthesized and studied for decades [1,2,4,5,6,14,15] and the efficacy of PEGylated and other liposomes as boron carriers has been repeatedly demonstrated [6,16,17,18,19,20]. However, although the formulation of BSH encapsulated in the inner aqueous phase of liposomes shows a certain antitumor effect in vivo, the amount of boron that can be delivered by liposomes to tumor tissue might be insufficient for human applications and such liposomes have not been applied clinically.

In this study, we developed boron-containing liposomes with features different from other previously synthesized liposomes for BNCT. The main difference is that the boron cluster (BSH) is modified via PEG and located at a distance from the liposome membrane, suggesting a very low effect on membrane stability. It has also been suggested that further inclusion of BSH may lead to membrane destabilization, which limits the options for further increasing BSH load in the liposomes. Therefore, our PBL liposomes, in which boron is modified in the outer aqueous phase rather than the inner aqueous phase, can greatly contribute to boron delivery. Furthermore, the amount of boron (BSH) for encapsulation can be optimized by adjusting its concentration and lipid composition as liposomes incorporating the maximum amount of boron for the inner and outer aqueous phases will efficiently shuttle boron into tumor cells for maximum BNCT effect.

Previous reports also show the synthesis of advanced liposomes combining diagnostic and therapeutic purposes [21,22,23]. While the inclusion of boron compounds, such as BSH in the inner water phase of our liposomes, can enhance the therapeutic effect of BNCT, encapsulation of other anticancer agents or diagnostic compounds in the inner aqueous phase can provide further theranostic combinations that can be realized with single drug application. Our synthesized liposomes, with options of increased boron delivery and the potential to include other compounds in the inner water phase, could provide advanced, synergistic options for therapeutic enhancement, and drive the development of new anticancer agents and boron carriers for BNCT.

The major limitation of this study is the lack of biological experiments. However, in this study, we designed and synthesized boron-containing liposomes as delivery vehicles for other compounds to be used or tested in BNCT. For biological experiments, additional boron or other compounds need to be incorporated into the inner part of the liposomes, depending on the design of the biological experiments, which was outside the scope of the present study. Therefore, we believe that such future experiments would elucidate the true utility of our reliable and versatile biological delivery system.

## 5. Conclusions

We developed a novel boron lipid, PBL, and novel boron liposomes using PBL in which the boron is present in the outer aqueous phase and does not affect the drug encapsulated in the inner aqueous phase. These liposomes are expected to have similar retention in the blood and tumor accumulation as PEG liposomes due to the coupling of BSH to the extremities of the PEG chains as well as the inclusion of boron compounds in the inner water phase to increase delivery payload. Additional encapsulation of other anticancer drugs in the inner aqueous phase will provide options for combination treatments within BNCT. PBL liposomes are expected to be promising candidates for new boron delivery agents for BNCT.

## Figures and Tables

**Table 1:** Characteristics of PBL liposomes.

Constituent PBL (%)	1	3	5
Incorporation efficiency (% ± SD)	65.6 ± 7.4	70.5 ± 1.7	36.4 ± 2.4
Loading content of PBL (µmol ± SD)	0.041 ± 0.01	0.107 ± 0.025	0.072 ± 0.039
Particle size (nm ± SD)	149.3 ± 5.9	173.8 ± 25.2	144.1 ± 3.2
Zeta potential (mV ± SD)	−27.0 ± 3.3	−46.5 ± 7.6	−45.4 ± 2.8

## Data Availability

The data presented in this study are available on request from the corresponding author.

## Appendix A

PBL liposomes (PBL content of 20%) were prepared using DSPC, cholesterol, and PBL (1:1:0.5, molar ratio) by using the lipid-film method and freeze-thaw method. Figure A1 shows the comparison between PBL content of 5% (dotted line) and 20% (solid line).
